# The Advent of Semi-Elective Lung Transplantation—Prolonged Static Cold Storage at 10°C

**DOI:** 10.3389/ti.2024.12310

**Published:** 2024-01-22

**Authors:** K. Hoetzenecker, A. Benazzo, S. Schwarz, S. Keshavjee, M. Cypel

**Affiliations:** ^1^ Department of Thoracic Surgery, Medical University of Vienna, Vienna, Austria; ^2^ Toronto Lung Transplant Program, Division of Thoracic Surgery, University Health Network, University of Toronto, Toronto, ON, Canada

**Keywords:** 10°C, lung transplantation, semi-elective, preservation, prolonged storage

## Abstract

Since the early days of clinical lung transplantation the preservation of donor organs has become a fairly standardized procedure and most centers do follow similar processes. This includes the use of low-potassium high dextran flush solutions and static cold storage (SCS) in a cooler filled with ice. Depending on the length of SCS, organs usually arrive at the recipient hospital at a temperature of 0°C–4°C. The question of the optimal storage temperature for donor lung preservation has been revisited as data from large animal experiments demonstrated that organs stored at 10°C experience less mitochondrial damage. Thus, prolonged cold ischemic times can be better tolerated at 10°C—even in pre-damaged organs. The clinical applicability of these findings was demonstrated in an international multi-center observational study including three high-volume lung transplant centers. Total clinical preservation times of up to 24 hrs have been successfully achieved in organs stored at 10°C without hampering primary organ function and short-term outcomes. Currently, a randomized-controlled trial (RCT) is recruiting patients with the aim to compare standard SCS on ice with prolonged SCS protocol at 10°C. If, as anticipated, this RCT confirms data from previous studies, lung transplantation could indeed become a semi-elective procedure.

## Introduction

Since the initiation of clinical lung transplantation, the procedure has always been defined as an emergent or acute operation, which requires teams to adhere to a strict timeline to minimize ischemic injury to the graft. There is currently a general consensus within the lung transplant community, that the length of graft preservation clinically should not exceed 6–8 h. This practice is reflected in large database analyses including the International Society for Heart and Lung Transplantation (ISHLT) Registry data. These studies have uniformly shown that short-term mortality increases with longer preservation time, with 30-day survival being significantly worse for ischemic times greater than 6 h [1]. Storage on ice has been considered the standard of care for preservation of donor lungs (and all organs for that matter) and has been implemented as such in lung transplant centers around the world. Storage on ice is intended to provide storage temperatures ranging between 0°C and 4°C, which is empirically considered clinically safe.

## Current Evidence in Donor Lung Preservation

The current practice in lung transplantation—including optimal donor lung preservation–is mostly based on empirical clinical experience and expert consensus. Due to the low number of procedures performed per year, it has often been considered difficult to provide robust data in the field of lung transplantation. Moreover, the significant heterogeneity among patients and center practice has represented a major hurdle to design multicenter prospective clinical trials. To the best of our knowledge, only two randomized controlled trials have been published in the field of donor lung preservation. Both of them compared an *ex-vivo* lung perfusion (EVLP) protocol with SCS. The INSPIRE trial [2] tested the Organ Care System in “standard” donor lungs. Before that, a single-center RCT was conducted by the Vienna Lung Transplant Program which compared the Toronto EVLP protocol to SCS, also in standard donor lungs [3]. Both trials showed comparable results of EVLP with the “simpler” and “cheaper” static preservation on ice. Therefore, EVLP did not replace SCS, but was introduced to clinical practice as a tool to evaluate lungs with a questionable quality and to optimize marginal donor lungs [4–8].

## Lung Preservation at 10°C

As early as 30 years ago, attempts were made to understand the effects of temperature on graft function and to optimize the temperature during donor lung preservation [9]. Subsequently this was confirmed in a study by Kayano et al., using a rat model, that reported an optimal storage temperature of 10°C [10]. Similar findings were observed in a large animal (canine) lung transplant study, published in 1992, comparing three different preservation temperatures [11]. Once again, lungs preserved at 10° degrees showed better oxygenation and decreased pulmonary vascular resistance. However, because of the lack of ability to accurately maintain organs at 10°C at the time (and incomplete understanding of the underlying biologic mechanisms), clinical concern that a temperature increase above this 10°C threshold could have deleterious effects on the lungs, the lower, more convenient target value of 4°C was widely adopted to provide a safety margin. As such, SCS on ice came to be defined as the “standard of care.”

One of the main disadvantages of SCS on ice is that the true temperature of the donor lung can significantly diverge from 4°C. A thermographic evaluation of donor lungs transported on ice showed that the surface temperature was non-homogenous and depending on preservation times ranged from 0.2°C to 10.6°C [12]. Consequently, temperature-controlled preservation devices, which use more accurate cool packs instead of ice cubes, have been developed.

### Rationale

The rationale underlying hypothermic organ storage is to reduce cellular metabolism, and thus maintain viability during the storage time with limited oxygen and nutrients. Since most of the deleterious effects of hypoxia are caused by simple biochemical reactions, it seemed reasonable to reduce the temperature close to 4°C in order to decrease enzyme activity in the donor organ. However, because this approach is non-selective, vital enzymes, such as Na+/K+ ATPase, are also affected in their function, leading to an ionic imbalance that can result in cell edema and damage [13]. In addition, intracellular calcium accumulation induces further cellular damage and the formation of reactive oxygen species can be promoted during cold ischemia [14]. In recent years, efforts have been made to optimize preservation strategies by revisiting the topic of optimal storage temperature of donor lungs. These efforts aimed to extend preservation times in order to increase the donor pool, optimize the immunological matching between donor and recipient and transition organ implantation from an urgent to a semi-elective procedure.

### Pre-Clinical Studies

**FIGURE 1:**
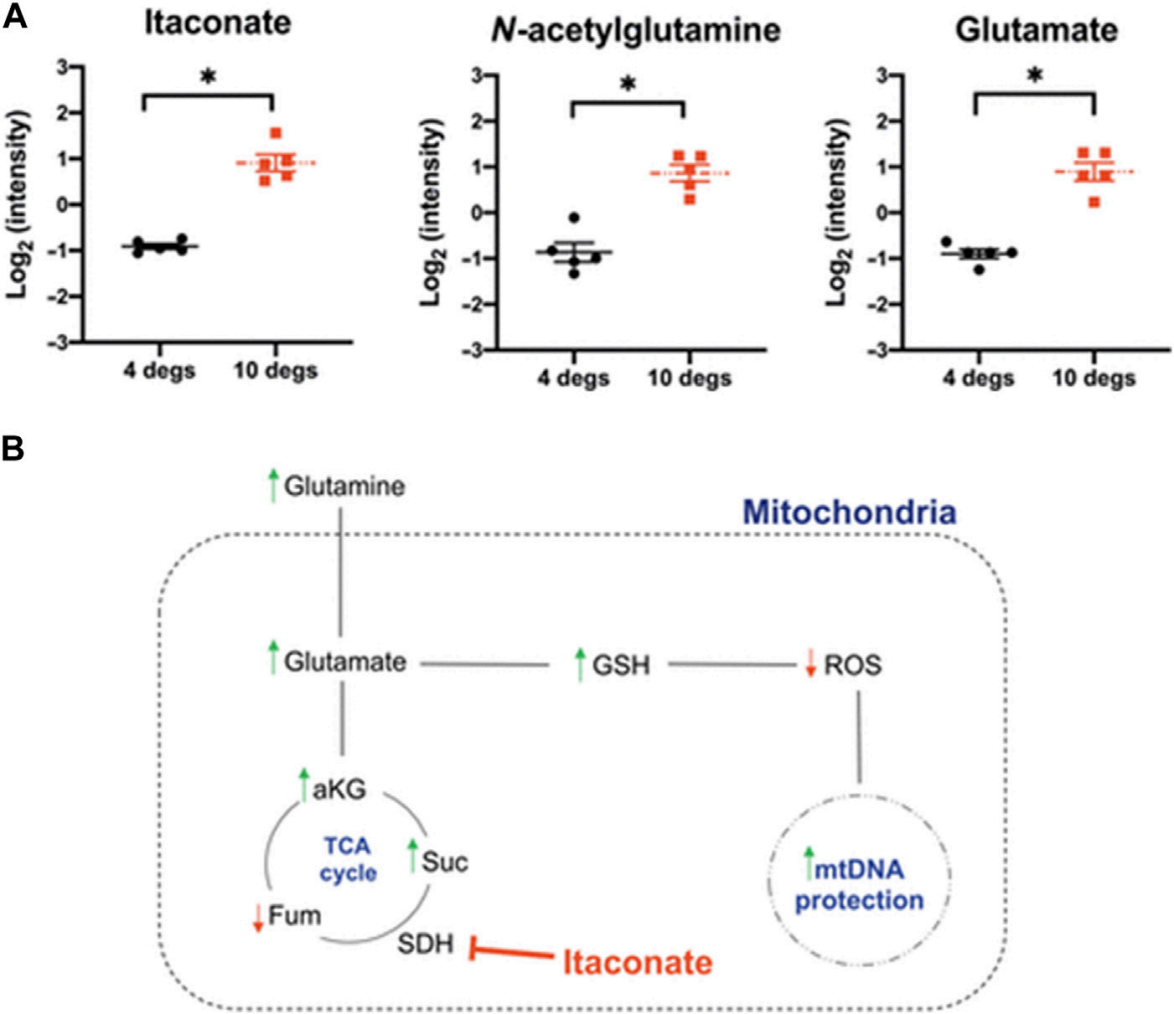
Levels of metabolites relevant for mitochondrial oxidative protection are displayed in **(A)**. **(B)** shows a scheme of how the tricarboxylic acid (TCA) cycle impacts mitochodrial health.

First, the feasibility of prolonged donor lung storage at 10°C was assessed in a large animal model. Porcine lungs stored for 36 h at 10°C showed lower airway pressures, had a better lung compliance and improved oxygenation after implantation as compared to lungs stored conventionally on ice [15]. Importantly, markers of mitochondrial injury were found to be lower in the study group, which provided a mechanistic insight into the benefit of 10°C storage (Figure 1). In a subsequent study, a possible recovery or regenerative effect of 10°C preservation was tested in a model of gastric acid aspiration injury [16]. Moderate lung damage was induced by intrabronchial instillation of gastric juice. Injured donor lungs were harvested and randomly assigned to storage for 12 h on ice or at 10°C. A third group consisted of immediate transplantation after only a short period of SCS on ice. A left single lung transplant was performed, followed by a 4-hour functional assessment. During reperfusion, lungs stored at 10°C showed significantly better oxygenation. Moreover, they had lower tissue levels of IL-1β after reperfusion, histologic evaluation demonstrated lower acute lung injury scores and significantly less apoptosis in the 10°C group. In all measured parameters, storage of lungs at 10°C for 12 h was associated with improved graft quality, even when compared to minimal cold ischemia on ice.

### Clinical Studies

These preclinical data were then translated to the clinical setting and a multicenter non-randomized clinical trial was designed. Three high-volume lung transplant centers—Toronto, Vienna, Madrid—recruited patients in a safety and feasibility study [17]. In this study, grafts from donors with cross-clamp times between 6:00 PM and 4:00 AM had an earliest possible implantation start time of 6:00 AM. Lungs were retrieved and transported using a traditional ice cooler. Upon arrival at the transplant hospital, the lungs were transferred to a temperature-controlled 10°C incubator (MYTEMP™65HC, Benchmark Scientific) and were stored until implantation. The primary outcome of this study was the incidence of Primary Graft Dysfunction (PGD) grade 3 at 72 h. Results were compared to a contemporary cohort of recipients, who received donor lungs that had been preserved by SCS on ice, using propensity score matching at a 1:2 ratio. Seventy patients were included in the study arm. Mean cold static preservation was significantly longer in the 10°C study group vs. matched controls for both the first and second implanted lung (Table 1). PGD grade 3 at 72 h was 5.7% in the study group vs. 9.3% in matched controls (*p* = 0.39). There were no differences in the need for post-op ECMO, median ICU length of stay (LOS) or median hospital LOS between the two groups. Also, one-year survival was similar between the two groups (*p* = 0.37) with a median follow-up time of 336 days [17].

**TABLE 1:** Post-transplant outcomes of pilot, prospective, multi-center, non-randomized clinical trial, adapted from Ali et.al. permission to reprint obtained [17].

Outcome	Study cohort (*n* = 70)	Matched controls (*n* = 140)	Difference (95% CI)
Incidence of PGD3 at 72 h, n (%)	4 (5.7)	13 (9.3)	−3.6 (−10.5, 5.3)
Recipient Vent time (hours), median (IQR)	49 (29, 82)	52 (27, 89)	−3 (−15, 7)
ICU LOS (days), median (IQR)	5 (3, 9)	5 (3, 12)	0 (−2, 1)
Hospital LOS (days), median (IQR)	25 (20, 40)	30 (20, 54)	−5 (−8, 2)
Post-LTx ECMO used, n (%)	5 (7.1)	13 (9.3)	−2.1 (−9.3, 7.2)
30-day survival, n (%)	70 (100)	135 (96.4)	3.6 (−2.0, 8.1)

LOS, length of stay; ICU, intensive care unit; PGD3, International Society for Heart and Lung Transplantation Primary Graft Dysfunction Grade 3; LTx, Lung transplantation; ECMO, Extracorporeal membrane oxygenation; Vent, Ventilation.

Based on the above findings, a multicenter, prospective, randomized-controlled trial was designed, to which recruitment officially started in May 2023 (NCT05898776). This study, involving 15 transplant centers worldwide, is designed as a non-inferiority study that will compare an extended preservation period (time from donor aortic cross-clamp to anesthesia start in the recipient hospital) of up to 12 hrs using a portable 10°C cooler (XPort, Traferox, Toronto, Canada) to conventional preservation (SCS on ice; time from donor aortic cross-clamp to anesthesia start in the recipient hospital of up to 6h). The results of this RCT will hopefully provide the evidence for changing the standard practice of donor lung preservation, which will in turn lead to significant flexibility in clinical lung preservation times.

## Advantages of Prolonged Storage at 10°C

### Avoid Night Time Transplantation

Multi-organ donation and the subsequent need to coordinate several organ procurement and implantation teams has shaped transplant medicine into an acute and challenging discipline. As all donor organs have different tolerances to cold ischemia and operating room capacity is often limited at procurement sites, multiorgan procurement is often performed in the evening or night time. As a result, implantation teams are often required to perform complex and exhausting procedures in critical recipients during night time hours. Of interest, several studies in the field of transplantation have linked night time procedures with worse clinical outcomes. It has been demonstrated that night time lung transplant recipients had a higher rate of postoperative complications than daytime recipients [18]. Similarly, it has been shown that night time liver transplant recipients have a 2-fold increased short-term mortality [19]. The possibility to prolong preservation times and thus render lung transplantation into a semi-elective and day time procedure has the potential to improve patient outcomes and as quality of life for transplant professionals, which would profoundly change current practice.

### Postpone Implantation for Logistic Reasons

The limited preservation time of grafts can create significant logistic problems for lung transplant centers. In this light, extending the time window of implantation by being able to store grafts at 10°C increases flexibility and offers several advantages: (i) scheduled elective cases can be finished by deliberately moving the implantation to the afternoon; (ii) surgically complex recipients can be transplanted during the day when the team can perform at its best, or the most experienced team is available; (iii) small and medium-sized lung transplant centers may be able to accept concurrent donor offers and safely implant the organs one after another. These logistical advantage has already in fact been successfully described by the Madrid transplant program, which accepted two parallel donors and postponed the most complex of the two cases until daytime, thus avoiding parallel surgery in the operating room overnight [20]. In addition, prolonged organ storage provides the opportunity to optimize a recipient preoperatively. As highly sensitized recipients are increasingly accepted by lung transplant programs, preoperative sensitization protocols including immunoadsorption or plasmapheresis could potentially be performed without time constraints. Finally, higher logistical flexibility can have a direct impact on the quality of life of transplant candidates. Patients on the waiting list who reside in remote areas may have the opportunity to remain in their hometown despite longer transport times without the need to relocate near the transplant center—a major social and economic advantage for them.

### Broader Geographic Distribution of Donor Organs

The shortage of donor lungs remains one of the biggest hurdles in clinical lung transplant practice. In Europe, North America and around the world, a large number of optimal donor offers are often declined, simply due to the long transport times involved. An easy-to-use, cost effective and reusable temperature-controlled device that ensures organ storage at 10°C for an extended period of time could fundamentally change our current practice. Based on the scientific evidence presented above, such a device could safely extend the geographic boundaries and facilitate a broader organ sharing.

Another interesting concept that is linked with increased preservation times, is fostering environmentally friendly transportation modes. Currently, the majority of donor lungs are transported by charter airplane flights [21]. Especially in Europe, where travelling distances are comparably short, most organs could be transported by commercial flights, car or train in the near future. This has the potential to significantly improve the carbon footprint of organ procurement and decrease costs [22].

### Reduce the Number of “False-Calls” for Recipients

“Dry runs” are common in lung transplantation with rates of up to 40 percent being reported in DCD donors [23]. Currently, recipients are immediately informed when an organ has been allocated to them and they usually have to promptly come to the hospital. “Dry runs” pose an enormous emotional burden to recipients and their families. Such false calls could be completely avoided by prolonged 10°C preservation as patients would only be informed when a lung has been finally accepted for transplantation.

## Future Directions of Organ Preservation

Donor lung preservation is one of the most studied topics of clinical lung transplantation. Most of recent research aims to either prolong preservation times or improve organ quality. We foresee and increasing role of *ex-vivo* lung perfusion with a constant improvement in perfusion and ventilation strategies. Several attempts have recently been made to optimize perfusion solutions in order to prolong EVLP times, i.e., adding nutrients or maintaining perfusate osmolality and pH [24, 25]. Another interesting concept might be the use of hypothermic *ex-vivo* lung perfusion [26]. HOPE is already routinely used in liver and kidney transplantation [27]. In addition, it has recently also been successfully tested in heart transplantation [28].

The other main perspective of *ex-vivo* lung perfusion is its role as a repair platform where reversible conditions of the donor organ can be treated. Promising data from animal study are already available. EVLP has been successfully used to reduce bacterial load [29], to reverse inflammatory damage related to aspiration [30], and to clear lungs from infections such as HCV [31]. In a subsequent step EVLP could be used to manipulate donor lungs and render them into ‘super organs’. Modulating immunogenicity by inducing IL-10 overexpression or cleaving surface antigens are possible approaches.

## Conclusion

Clinical donor lung preservation will significantly change in the future. Storage of grafts at 10°C will have a considerable impact on transplant programs around the world by extending acceptable and safe preservation times. This will shift in our clinical practice towards an unprecedented semi-elective transplantation practice with numerous beneficial effects.
